# The hypertension cascade of care in the midst of conflict: the case of the Gaza Strip

**DOI:** 10.1038/s41371-022-00783-w

**Published:** 2022-12-12

**Authors:** Bassam A. Abu Hamad, Zeina Jamaluddine, Gloria Safadi, Marie-Elizabeth Ragi, Raeda El Sayed Ahmad, Eszter P. Vamos, Sanjay Basu, John S. Yudkin, Mohammed Jawad, Christopher Millett, Hala Ghattas

**Affiliations:** 1 School of Public Health, Al-Quds University, Gaza, Palestine; 2 Center for Research on Population and Health, American University of Beirut, Beirut, Lebanon; 3 Faculty of Epidemiology and Population Health, London School of Hygiene and Tropical Medicine, London, UK; 4 Public Health Policy Evaluation Unit, School of Public Health, Imperial College London, London, UK; 5 Research and Development, Waymark, San Francisco, CA USA; 6 Institute of Cardiovascular Science, Division of Medicine, University College London, London, UK; 7 Comprehensive Health Research Centre and Public Health Research Centre, National School of Public Health, NOVA University, Lisbon, Portugal

**Keywords:** Health care, Risk factors, Hypertension, Diagnosis

## Abstract

Although hypertension constitutes a substantial burden in conflict-affected areas, little is known about its prevalence, control, and management in Gaza. This study aims to estimate the prevalence and correlates of hypertension, its diagnosis and control among adults in Gaza. We conducted a representative, cross-sectional, anonymous, household survey of 4576 persons older than 40 years in Gaza in mid-2020. Data were collected through face-to-face interviews, anthropometric, and blood pressure measurements. Hypertension was defined in anyone with an average systolic blood pressure ≥140 mmHg or average diastolic blood pressure ≥90 mmHg from two consecutive readings or a hypertension diagnosis. The mean age of participants was 56.9 ± 10.5 years, 54.0% were female and 68.5% were Palestinian refugees. The prevalence of hypertension was 56.5%, of whom 71.5% had been diagnosed. Hypertension was significantly higher among older participants, refugees, ex-smokers, those who were overweight or obese, and had other co-morbidities including mental illnesses. Two-thirds (68.3%) of those with hypertension were on treatment with one in three (35.6%) having their hypertension controlled. Having controlled hypertension was significantly higher in females, those receiving all medications for high blood pressure and those who never or rarely added salt to food. Investing in comprehensive but cost-effective initiatives that strengthen the prevention, early detection and timely treatment of hypertension in conflict settings is critical. It is essential to better understand the underlying barriers behind the lack of control and develop multi-sectoral programs to address these barriers.

## Introduction

Evidence suggests that noncommunicable diseases (NCDs) have replaced infectious diseases as leading causes of morbidity and mortality in protracted conflict settings [1, 2]. Political instability, high stress levels, forced migration, and health system prioritization of lifesaving interventions can hamper NCD management and prevention [1]. Socioeconomic vulnerabilities such as overcrowding, low quality housing, and poor infrastructure can impede physical activity and access to nutritious food and also increase psychological distress, while coping strategies may include increased smoking and alcohol use [2].

The Gaza Strip is a narrow sliver of land (45 km long) between Israel, Egypt, and the Mediterranean Sea, home to around 2 million people, making it one of the world’s most densely populated areas [3]. According to the Palestinian Central Bureau of Statistics (PCBS) two thirds of Gaza’s inhabitants (66%) are refugees [3], most having been forcibly displaced from their original villages and cities following the Arab–Israeli conflict of 1948. Around 40% of the refugees in Gaza live in one of the eight refugee camps [3] but all refugees have the same civil legal status and entitlements as non-refugees, including access to social services, employment, and other rights. However, expropriation of land has created a condition of dispossession that has further compromised Palestinians’ abilities to withstand the deliberate de-development strategy pursued by Israel, through punitive economic and military policies. Israel still has overall sovereignty of Gaza, controlling its borders, economy, movement of goods and people, electricity, communications, and security—the key aspects of Palestinians’ lives [4].

The key socioeconomic and environmental determinants of health for the entire Gaza population have been negatively affected by the ongoing conflict which has continued for more than seven decades. This includes 16 years of strict blockade and economic collapse, which has increased health-related vulnerabilities. Gaza suffers from a high NCD burden [5] in the context of occupation, poverty, profound psychosocial distress, and limited access to health services. Nearly half of the households in Gaza are food insecure [3, 6], and 80% of the population is highly reliant on external assistance, particularly food. The United Nations Office for the Coordination of Humanitarian Affairs has repeatedly described the situation in Gaza as a chronic emergency and a protracted human dignity crisis [7].

The Gazan healthcare system is largely fragmented, poorly governed and under-funded, composed of mixed public/private/humanitarian services provided by the Ministry of Health, the United Nations Relief and Works Agency for Palestine Refugees in the Near East (UNRWA), nongovernmental organizations, and private for-profit operators [8]. While the Ministry of Health serves medically insured refugees and non-refugees, UNRWA, provides social services including health to the refugee population in Gaza. Nearly 40% of total health expenditure in Gaza is out-of-pocket [8]. Despite that health insurance coverage is widespread (more than 90% of households), still it does not meet people’s needs; few medicines are covered by insurance (if available at all), and there are limited specialist services and long waiting lists [4]. While people are ordinarily able to access basic health services, access becomes challenging during renewed outbreaks of conflict, and access to advanced services outside Gaza (such as radiotherapy, advanced cardiac and neurosurgery) remains limited [4]. Despite the limited available resources, inefficiencies in the health care system are still common and resources are unnecessarily wasted due to duplication, fragmentation and ineffective coordination including weak referral systems among service providers [8].

Hypertension is one of the most important risk factors for NCDs, and a recent systematic review found that hypertension constitutes a substantial burden in humanitarian settings [2]. However, its prevalence, risk factors and management in Gaza is poorly investigated. One survey conducted in 2017 reported that 28.4% of the adult population in Gaza had hypertension, but this study was limited by its non-representative sample and suboptimal methodology for measuring blood pressure [9]. Furthermore, a broader understanding of the hypertension cascade of care in Gaza is urgently needed to identify barriers and quantify unmet needs to diagnosis, treatment, and control. We therefore aimed to estimate the prevalence and correlates of hypertension among adults in Gaza, including an assessment of sociodemographic inequalities across the cascade of care.

## Subjects and methods

### Design and sampling

We conducted a representative, cross-sectional, anonymous, household survey of persons older than 40 years living in all five governorates of the Gaza Strip. We used the 2017 Population and Housing Census as the sampling frame to select enumeration areas (the primary sampling units) from each sampling stratum (North Gaza, Gaza, Dier Al Balah, Khan Yunis, and Rafah governorates) using a systematic cluster random sampling method. We calculated the sample size as 4520 participants from 2443 households based on an estimated prevalence of coronary artery disease (11.3%) [9], a response rate of 90.0%, and design effect of 1.5.

We selected 163 clusters proportionate to the size of the population in the five governorates. In each cluster, the PCBS provided a starting point and data collectors approached every 10th household until 15 were sampled. In each household, we interviewed one eligible male and one eligible female older than 40 years old. If multiple eligible participants of the same sex lived in the same household, we randomly selected one based on a Kish selection grid. We replaced non-respondent or ineligible households using the same selection criteria.

We trained interviewers for four days prior to data collection between March and July 2020, which was delayed for eight weeks due to COVID-19 lockdown measures. A pair of interviewers visited each household and obtained verbal informed consent prior to data collection. We checked participants’ responses for completeness daily, included built-in quality control measures into our survey, and revisited subset of 220 households to verify responses. Where interviewers identified urgent clinical support required for participants, a referral pathway was put in place for to link them to accredited service providers. The study was approved by the Imperial College Research Ethics Committee (20IC5733), the American University of Beirut Institutional Review Board, and the Gaza Helsinki Committee (PHRC/HC/483/19).

### Survey

We adapted our survey from previously validated surveys, prioritizing those conducted on Palestinian or Arab populations. Briefly, we collected information on participants’ demographics, social assistance, food insecurity (based on the Food Insecurity Experience Scale [10], mental wellbeing (based on the General Health Questionnaire-12 [11] and self-rated health. We asked diagnostic, management, and health service utilization questions about diabetes, hypertension, raised total cholesterol, cardiovascular diseases, chronic respiratory diseases, and cancer [12]. We assessed physical activity (based on the International Physical Activity Questionnaire short form [13], salt intake, tobacco use (based on the World Health Organization-WHO Steps Survey [14] and a detailed semi-quantitative food frequency questionnaire [14, 15] as key NCD risk factors.

We measured height to the nearest centimeter using a Seca 217 stadiometer (Seca GmbH & Co, Hamburg, Germany) and weight to the nearest 10 g using an adult, portable electronic Seca 876 scale. We measured waist circumference mid-way between the lateral lower rib margin and the iliac crest using a Seca 201 meter. We measured blood pressure using the Omron M3 automatic upper arm blood pressure device (Omron Healthcare Co., Ltd. Kyoto, Japan). We developed written guidelines on blood pressure measurement which were used to train data collectors. We ensured participants were at rest for at least 10 min, had abstained from exercising, smoking or drinking coffee for at least 30 min prior to measurement, had removed clothes that could constrict blood vessels, had an empty bladder, and sat straight in a relaxed position with uncrossed legs with their left arm at the heart level. We also ensured an appropriate size positioned one inch above the elbow, which the forearm supported on a table with an upward palm upward, and at least five minutes interval between the two blood pressure readings.

We took the average weight, height, and waist circumference from two consecutive readings. For both systolic blood pressure (SBP) and diastolic blood pressure (DBP), we also relied on two readings. Where there were inconsistencies between the two readings (more than 5 mmHg), we performed a third measurement. For each participant, average SBP and DBP was computed as the average of the recorded readings. The average blood pressure measures were those computed from all readings (two consecutive, or three if inconsistencies) performed. We excluded pregnant women (*n* = 12) and participants who had an amputation (*n* = 26) from anthropometric measurements.

### Measures

Three outcome measures, reflecting the cascade of care, were total hypertension, diagnosed hypertension, and controlled hypertension. Participants were classified as hypertensive if having an average SBP ≥ 140 mmHg or average DBP ≥ 90 mmHg or a hypertension diagnosis. We defined diagnosed hypertension as anyone being informed by a doctor or other health worker that they had hypertension, or if they had taken medication for hypertension as prescribed by their doctor or another health care worker in the previous 2 weeks. We defined treated hypertension as taking, within the past 2 weeks, prescribed antihypertensive medications. We defined controlled hypertension as anyone with diagnosed hypertension with <140 mmHg systolic and <90 mmHg diastolic blood pressure readings.

Sociodemographic independent variables included age (10 year brackets), refugee status (refugee/non-refugee), sex (male/female), governorate (North Gaza/Gaza/Deir Al Balah/Khan Younis/Rafah), education (basic/intermediate/secondary or higher), wealth quintiles based on household assets, income per capita, household size, crowding index (<1/2–3/>3 people per room), locality (camp/non-camp), marital status (not married/engaged or married), employment (yes/no), food insecurity (secure [score 0–3]/mild-to-moderately insecure [4–6]/severely insecure [7,8]), health insurance (yes/no), and past-year social assistance (yes/no).

Risk factor independent variables included physical activity (low/moderate/high, based on the metabolic equivalent minutes per week), tobacco (never/current/quit), the Dietary Interventions to Stop Hypertension (DASH) index [16], and salt addition to food (always/often/sometimes/rarely/never). Independent variables included the presence of a co-morbidity (defined as the presence of at least one disease: diabetes (yes/no), elevated cholesterol (yes/no), heart attack/chest pain (yes/no), stroke (yes/no), cancer (yes/no) pulmonary disease (yes/no), body mass index (underweight [<18.5 kg/m^2^]/normal [18.5–24.9]/overweight [25.0–29.9]/obese [>30.0]), mental wellbeing (minimal mental illness or psychosocial distress [score <5]/mild [6,7]/moderate-severe [>7]), self-rated health (not good or not good at all/half-half/good or very good) [17].

### Statistical analysis

We performed descriptive statistics to characterize our sample, stratified by sex, and to present the cascade of hypertension care. We used multiple logistic regression models to assess the crude and adjusted associations between independent variables and each outcome (total hypertension, diagnosed hypertension, controlled hypertension). Variables were selected based on potential risk factors, previously identified in the literature, related to hypertension regardless of their statistical association with the outcome [18]. We presented models clustered at the household level. We presented odds ratios, their 95% confidence interval (95% CI) and *p* value. We also presented the effect modification of age and sex on the prevalence, diagnosis, and control of hypertension. We calculated variance inflation factor to test for multicollinearity. Around 5% of data were excluded from the models due to missing data. We ran the models with and without missing data and found no differences in our findings so present models with missing data. We used Stata 15 (StataCorp) and Statistical Package for Social Sciences (SPSS) 25 (IBM) for these analyses.

## Results

### Sample characteristics

We obtained a 96.6% response rate and included 4576 participants, of whom 54.0% were females and 68.5% were refugees. Sample characteristics stratified by sex and having versus not having hypertension are detailed in Tables 1A and 1B. The mean age of participants was 56.9 ± 10.5 years, most resided in Gaza (34.4%) and in urban/semi-urban localities (85.7%), 41.6% had secondary or higher educational attainment, and 82.1% were unemployed. Males were significantly older, had higher educational attainment and were more likely to be employed. At the household level, the median monthly income was 804.7 New Israeli Shekels (NIS) (interquartile range [IQR] 516.7–1400.0 NIS) (USD 230.13, IQR 147.85–400.64) and mean household size was 6.27 ± 3.18. Only 42.9% of participants were food secure (Table 1A). Because of the harsh economic status, 77.1% of participants received some sort of assistance in the past 12 months. Most participants had health insurance (83.8%). Physical inactivity was prominent, 52.5% of male participants and 70.2% of female participants reported practicing low physical activity.

**Table 1:** **A.** Sociodemographic characteristics and prevalence of co-morbidities in Gaza by sex. **B.** Sociodemographic characteristics and prevalence of co-morbidities in Gaza by the presence versus absence of hypertension.

A.							
Characteristics/variables	Total		Male		Female		
	*N* (4576)	% or median [IQR]	*N* (2103)	% or median (IQR)	*N* (2473)	% or median (IQR)	*P* value
Socio-demographic							
Age categories (*N* = 4576)							
40–49	1289	28.2%	419	19.9%	870	35.2%	<0.001^a^
50–59	1575	34.4%	781	37.1%	794	32.1%	
60–69	1091	23.8%	561	26.7%	530	21.4%	
70 plus	621	13.6%	342	16.3%	279	11.3%	
Refugee status (*N* = 4576)							
Refugee	3136	68.5%	1427	67.9%	1709	69.1%	0.364^a^
Non refugee	1440	31.5%	676	32.1%	764	30.9%	
Sex of the head of the household (*N* = 4576)							
Male headed household	4110	89.8%	2093	99.5%	2017	81.6%	<0.001^a^
Female headed household	466	10.2%	10	0.5%	456	18.4%	
Marital status (*N* = 4576)							
Not married	446	9.8%	32	1.5%	414	16.7%	<0.001^a^
Engaged/Married	4130	90.2%	2071	98.5%	2059	83.3%	
Governorates (*N* = 4576)							
North Gaza	827	18.1%	382	18.2%	445	17.9%	0.887^a^
Gaza	1576	34.4%	715	34.0%	861	34.8%	
Deir Al Balah	721	15.8%	338	16.1%	383	15.5%	
Khan Yunis	875	19.1%	395	18.8%	480	19.5%	
Rafah	577	12.6%	273	12.9%	304	12.3%	
Locality (*N* = 4576)							
Urban	3922	85.7%	1806	85.9%	2116	85.6%	0.763^a^
Camp	654	14.3%	297	14.1%	357	14.4%	
Education (*N* = 4576)							
Basic	637	13.9%	240	11.4%	397	16.1%	<0.001^a^
Intermediate	2034	44.5%	906	43.1%	1128	45.6%	
Secondary Plus	1905	41.6%	957	45.5%	948	38.3%	
Employment status (*N* = 4574)							
Employed	817	17.9%	678	32.3%	139	5.6%	<0.001^a^
Unemployed	3757	82.1%	1424	67.7%	2333	94.4%	
Crowding index (*N* = 4555)							
<=1 person/room	3004	65.9%	1353	64.7%	1651	67.0%	0.224^a^
2–3 people/room	1138	24.9%	546	26.1%	592	24.1%	
>3 people/room	413	9.2%	193	9.2%	220	8.9%	
Household income (*N* = 4252)							
Median household income (NIS^c^)	4252	804.17 [516.67–1400.0]	1963	850 [560.0–1500.0]	2289	800 [500.0–1400.0]	0.140^b^
Median household income (USD)	4252	230.13 [147.85–400.64]	1963	243.24 [160.26–429.26]	2289	228.94 [143.09–400.64]	0.140^b^
FIES (*N* = 4568)							
Food secure	1961	42.9%	879	41.8%	1082	43.8%	0.531^a^
Mild to moderately food insecure	2348	51.4%	1094	52.3%	1254	50.7%	
Severely food insecure	259	5.7%	126	5.9%	133	5.5%	
Health insurance (*N* = 4571)							
No	740	16.2%	343	16.3%	397	16.2%	0.939^a^
Yes	3831	83.8%	1758	83.7%	2073	83.8%	
Assistance (*N* = 4576)							
Yes (any assistance)	3526	77.1%	1617	76.9%	1909	77.2%	0.808^a^
None	1050	22.9%	486	23.1%	564	22.8%	
Physical activity (*N* = 4576)							
Low	2839	62.0%	1104	52.5%	1735	70.2%	<0.001^a^
Moderate	1212	26.5%	774	36.8%	438	17.7%	
High	525	11.5%	225	10.7%	300	12.1%	
Other co-morbidities (*N* = 4486)							
No	2386	53.2%	1077	52.3%	1309	54.0%	0.249^a^
Yes	2100	46.8%	984	47.7%	1116	46.0%	
Mental illness (*N* = 4545)							
Minimum	3269	71.9%	1515	72.3%	1754	71.5%	0.091^a^
Mild	635	14.0%	269	12.9%	366	14.9%	
Moderate-severe	641	14.1%	309	14.8%	332	13.6%	
Self-rated heath (*N* = 4574)							
Very good/good	2533	55.4%	1189	56.5%	1344	54.4%	0.026^a^
Half/half	1229	26.9%	525	25.0%	704	28.5%	
Not good/not good at all	812	17.7%	388	18.5%	424	17.1%	

B.							
Characteristics/variables	Total		Hypertensive		Non-hypertensive		
	*N* (4576)	% or median [IQR]	*N* (2586)	% or median (IQR)	*N* (1990)	% or median (IQR)	*P* value
Socio-demographic							
Age categories (*N* = 4576)							
40–49	1289	28.2%	458	17.7%	831	41.8%	<0.001^a^
50–59	1575	34.4%	886	34.3%	689	34.6%	
60–69	1091	23.8%	789	30.5%	302	15.2%	
70 plus	621	13.6%	453	17.5%	168	8.4%	
Refugee status (*N* = 4576)							
Refugee	3136	68.5%	1820	70.4%	1316	66.1%	0.002^a^
Non refugee	1440	31.5%	766	29.6%	674	33.9%	
Sex (*N* = 4576)							
Male	2103	46.0%	1199	46.4%	904	45.4%	0.528^a^
Female	2473	54.0%	1387	53.6%	1086	54.6%	
Sex of the head of the household (*N* = 4576)							
Male	4110	89.8%	2319	89.7%	1791	90.00%	0.719^a^
Female	466	10.2%	267	10.3%	199	10.00%	
Marital status (*N* = 4576)							
Not married	446	9.7%	273	10.6%	173	8.7%	0.035^a^
Engaged/Married	4130	90.3%	2313	89.4%	1817	91.3%	
Governorates (*N* = 4,576)							
North Gaza	827	18.1%	460	17.8%	367	18.4%	<0.001^a^
Gaza	1576	34.4%	924	35.7%	652	32.8%	
Deir Al Balah	721	15.8%	436	16.7%	285	14.4%	
Khan Yunis	875	19.1%	425	16.5%	450	22.7%	
Rafah	577	12.6%	341	13.3%	236	11.7%	
Locality (*N* = 4576)							
Urban	3922	85.7%	2191	84.7%	1731	87.0%	0.030^a^
Camp	654	14.3%	395	15.3%	259	13.0%	
Education (*N* = 4576)							
Basic	637	13.9%	430	16.6%	207	10.4%	<0.001^a^
Intermediate	2034	44.5%	1145	44.3%	889	44.7%	
Secondary Plus	1905	41.6%	1011	39.1%	894	44.9%	
Employment status (*N* = 4574)							
Employed	817	17.9%	377	14.6%	440	22.1%	<0.001^a^
Unemployed	3757	82.1%	2208	85.4%	1549	77.9%	
Crowding index (*N* = 4555)							
<=1 person/room	3004	66.0%	1749	67.9%	1255	63.4%	0.002^a^
2–3 people/room	1138	25.0%	618	24.0%	520	26.3%	
>3 people/room	413	9.0%	208	8.1%	205	10.3%	
Household income (*N* = 4252)							
Median household income (NIS^c^)	4252	804.17 [516.67–1400.0]	2393	800.0 [500.0–1400.0]	1859	912.0 [600.0–1500.0]	0.075^b^
Median household income (USD)	4252	230.13 [147.85–400.64]	2393	228.94 [143.09–400.64]	1859	260.99 [171.70–429.26]	0.075^b^
FIES (*N* = 4568)							
Food secure	1961	42.9%	1099	42.5%	862	43.4%	0.852^a^
Mild to moderately food insecure	2348	51.4%	1336	51.8%	1012	50.9%	
Severely food insecure	259	5.7%	146	5.7%	113	5.7%	
Health insurance (*N* = 4571)							
No	740	16.2%	397	15.4%	343	17.3%	0.087^a^
Yes	3831	83.8%	2186	84.6%	1645	82.7%	
Assistance (*N* = 4576)							
Yes (any assistance)	3526	77.1%	2030	78.5%	1496	75.2%	0.008^a^
None	1050	22.9%	556	21.5%	494	24.8%	
Physical activity (*N* = 4576)							
Low	2839	62.0%	1715	66.3%	1124	56.5%	<0.001^a^
Moderate	1212	26.5%	611	23.6%	601	30.2%	
High	525	11.5%	260	10.1%	265	13.3%	
Other co-morbidities (*N* = 4486)							
No	2386	53.2%	985	38.7%	1401	72.2%	0.249^a^
Yes	2100	46.8%	1561	61.3%	539	27.8%	
Mental illness (*N* = 4545)							
Minimum	3269	71.9%	1768	68.9%	1501	75.9%	<0.001^a^
Mild	635	14.0%	384	15.0%	251	12.7%	
Moderate-severe	641	14.1%	414	16.1%	227	11.4%	
Self-rated heath (*N* = 4574)							
Very good/good	2533	55.4%	1214	47.0%	1319	66.4%	<0.001^a^
Half/half	1229	26.9%	787	30.4%	442	22.2%	
Not good/not good at all	812	17.7%	585	22.6%	227	11.4%	

^a^Chi square test for categorical variables.

^b^T-test for continuous variables.

^c^1 New Israeli Shekel (NIS) = 0.28617 United States Dollar (USD) from May through July 2020.

Self-reported prevalence of other NCD co-morbidities was 46.8% (Table 1A). Table 2 also shows that 25.8% of all participants reported having diabetes and 22.3% reported having elevated cholesterol level. Significant differences were found between males and females regarding self-reported heart attack, stroke, and cancer (Table 2). Male participants were more likely to report having heart attacks (13.3%) and strokes (5.9%) while less likely to report cancer (1.7%) as opposed to their female counterparts (Table 2).

**Table 2:** Prevalence of self-reported co-morbidities in Gaza.

Morbidity type	Total		Male		Female		*P* value
	*N* 4576	%	*N* 2103	%	*N* 2473	%	
Diabetes (*N* = 4550)							
No	3378	74.2%	1560	74.8%	1818	73.8%	0.412
Yes	1172	25.8%	525	25.2%	647	26.2%	
Elevated Cholesterol (*N* = 4495)							
No	3494	77.7%	1619	78.3%	1875	77.3%	0.439
Yes	1001	22.3%	450	21.7%	551	22.7%	
Heart Attack/Chest Pain (*N* = 4576)							
No	4152	90.7%	1824	86.7%	2328	94.1%	0.001
Yes	424	9.3%	279	13.3%	145	5.9%	
Stroke (*N* = 4576)							
No	4367	95.4%	1979	94.1%	2388	96.6%	0.001
Yes	209	4.6%	124	5.9%	85	3.4%	
Cancer (*N* = 4576)							
No	4474	97.8%	2068	98.3%	2406	97.3%	0.017
Yes	102	2.2%	35	1.7%	67	2.7%	
Pulmonary Disease (*N* = 4576)							
No	4127	90.2%	1891	89.9%	2236	90.4%	0.573
Yes	449	9.8%	212	10.1%	237	9.6%	

### Hypertension cascade

**Fig. 1:**
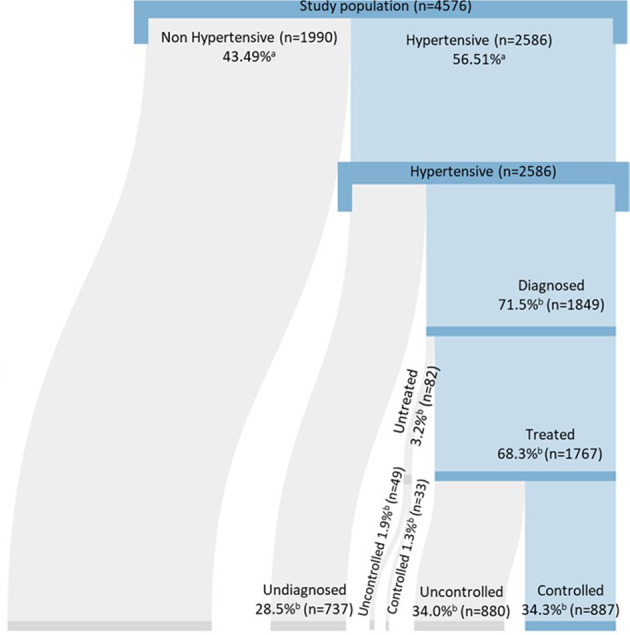
Hypertension cascade of care in Gaza. Percentages are indicated with as denominator the ^a^total study population (*n* = 4576) and ^b^hypertensive population (*n* = 2586).

The hypertension cascade of care is illustrated in Fig. 1. The total prevalence of hypertension in the study population (systolic blood pressure ≥140 mmHg or diastolic blood pressure ≥90 mmHg) was 56.5% (*n* = 2586), of whom 71.5% (*n* = 1849) had been diagnosed, 68.3% (*n* = 1767) of all those with hypertension were on treatment, and 35.6% (*n* = 920) were controlled. Indeed, 34.3% (*n* = 887) of treated hypertensive were controlled, while 1.3% (*n* = 33) of the untreated were controlled. Of the diagnosed with hypertension (*n* = 1849), the majority were on treatment (*n* = 1767) reaching a high proportion of 95.6%, and half of those who were diagnosed and under treatment had their blood pressure controlled (*n* = 887). However, using the cut off points of ≥130/≥80 mmHg for diagnosis of hypertension, the 56.5% total prevalence observed in the study population is substantially increased to 79.5%; 77.4% among females and 81.98% among males.

The crude and adjusted odds of having hypertension are shown in Table 3. In the adjusted model, significantly higher odds of having hypertension were found among those who were older, refugees, had lower educational attainment, were ex-smokers, overweight or obese, had other co-morbidities, and had mild or moderate-to-severe mental illness. Geographical inequalities were also evident; living in Gaza governorate was associated with higher odds of hypertension than living in Khan Yunis governorate. All three age groups (50–59 years, 60–69 years, 70+ plus) had higher odds of hypertension than those aged 40–49 years and this relationship appeared stronger with the advances in age.

**Table 3:** Correlates of hypertension prevalence in Gaza.

Variable	Categories	OR (95% CI) (*N* = 4576)	Adjusted OR (95% CI) (*N* = 4315)
Age	40–49 years	1.00	1.00
	50–59 years	2.33 (2.01–2.71)***	1.96 (1.65–2.33)***
	60–69 years	4.74 (3.98–5.64))***	3.49 (2.88–4.24)***
	≥70 years	4.89 (3.95–6.06)***	3.60 (2.78–4.66)***
Refugee status	Non-refugee	1.00	1.00
	Refugee	1.22 (1.07–1.38)**	1.20 (1.03–1.40)*
Sex	Male	1.00	1.00
	Female	0.96 (0.86–1.08)	0.87 (0.73–1.05)
Governorate	Khan Younes	1.00	1.00
	North Gaza	1.33 (1.09–1.62)**	1.32 (1.04–1.66)*
	Gaza	1.50 (1.26–1.79)***	1.41 (1.16–1.71)**
	Rafah	1.53 (1.23–1.91)***	1.67 (1.30–2.15)***
	Deir Al Balah	1.62 (1.31–2.00)***	1.56 (1.23–1.98)***
Education	Basic	1.00	1.00
	Intermediate	0.62 (0.51–0.75)***	0.91 (0.72–1.14)
	Secondary or higher	0.54 (0.45–0.66)***	0.77 (0.61–0.98)*
Wealth quintiles	Poorest	1.00	-
	2	0.81 (0.66–0.97)*	-
	3	0.90 (0.73–1.09)	-
	4	1.01 (0.83–1.23)	-
	Richest	0.97 (0.79–1.19)	-
Crowding Index	<= 1 person/room	1.00	-
	2–3 people/room	0.85 (0.74–0.99)*	-
	>3 people/room	0.73 (0.59–0.90)**	-
Locality	Non-camp	1.00	-
	Camp	1.20 (1.01–1.43)*	-
Marital status	Not married	1.00	-
	Engaged/Married	0.80 (0.66–0.98)*	-
Employment	No	1.00	-
	Yes	0.60 (0.52–0.70)***	-
Food insecurity	None	1.00	-
	Mild-to-moderate	1.04 (0.91–1.17)	-
	Severe	1.01 (0.77–1.33)	-
Health insurance	No	1.00	-
	Yes	1.15 (0.97–1.34)	-
Assistance	No	1.00	-
	Yes	1.21 (1.04–1.39)*	-
Physical activity	Low	1.00	-
	Moderate	0.67 (0.58–0.76)***	-
	High	0.64 (0.53–0.78)***	-
Tobacco	Never	1.00	1.00
	Current	0.74 (0.63–0.86)***	0.98 (0.78–1.23)
	Quit	1.69 (1.40–2.05)***	1.28 (0.99–1.66)
DASH Index	-	1.04 (1.02–1.05)***	-
Add salt/salty sauces to food	Yes	1.00	-
	No	1.21 (1.01–1.44)*	-
Body Mass Index	Normal	1.00	1.00
	Overweight	1.74 (1.43–2.12)***	1.89 (1.50–2.39)***
	Obese	3.45 (2.87–4.16)***	3.75 (2.98–4.71)***
Other co-morbidities	No	1.00	1.00
	Yes	4.12 (3.62–4.68)***	2.78 (2.42–3.20)***
Mental illness	Minimum	1.00	1.00
	Mild	1.30 (1.09–1.55)**	1.25 (1.02–1.53)*
	Moderate-severe	1.55 (1.30–1.85)***	1.31 (1.06–1.62)*
Self-rated health	Very good/good	1.00	-
	Half/half	1.93 (1.68–2.23)***	-
	Not good/not good at all	2.80 (2.36–3.32)***	-

In the adjusted model, the variables adjusted for are those with specified adjusted OR value.

*OR* odds ratio, 95% *CI* confidence interval, *DASH* Dietary Interventions to Stop Hypertension.

*p < 0.05, **p < 0.01, ***p < 0.001.

The crude and adjusted odds of having diagnosed hypertension compared to those with undiagnosed hypertension are shown in Table 4. In the adjusted model, significantly higher odds of diagnosed hypertension were found among those who were older, female, refugees, obese, had other co-morbidities, were engaged or married, had low physical activity, had moderate-to-severe mental illness, and who never added salt to food. Associations by age groups again showed a dose-dependent relationship.

**Table 4:** Correlates of hypertension diagnosis in Gaza.

Variable	Categories	OR (95% CI) (*N* = 2581)	Adjusted OR (95% CI) (*N* = 2434)
Age	40–49 years	1.00	1.00
	50–59 years	1.89 (1.49–2.39)***	1.65 (1.25–2.17)***
	60–69 years	2.87 (2.23–3.70)***	2.11 (1.55–2.87)***
	≥70 years	3.73 (2.75–5.06)***	3.05 (2.09–4.44)***
Refugee status	Non-refugee	1.00	1.00
	Refugee	1.38 (1.14–1.66)**	1.23 (1.02–1.59)*
Sex	Male	1.00	1.00
	Female	1.56 (1.32–1.85)***	1.65 (1.30–2.09)***
Governorate	Khan Yunis	1.00	-
	North Gaza	1.07 (0.80–1.43)	-
	Gaza	1.34 (1.04–1.74)*	-
	Rafah	1.44 (1.05–1.97)*	-
	Deir Al Balah	1.26 (0.93–1.71)	-
Education	Basic	1.00	-
	Intermediate	0.73 (0.56–0.95)*	-
	Secondary or higher	0.66 (0.51–0.86)**	-
Wealth quintiles	Poorest	1.00	-
	2	0.99 (0.74–1.31)	-
	3	1.15 (0.86–1.53)	-
	4	1.30 (0.97–1.73)	-
	Richest	1.27 (0.95–1.68)	-
Crowding Index	<= 1 person/room	1.00	-
	2–3 people/room	0.69 (0.57–0.85)***	-
	>3 people/room	0.62 (0.46–0.83)**	-
Locality	Non-camp	1.00	-
	Camp	1.09 (0.85–1.40)	-
Marital status	Not married	1.00	1.00
	Engaged/Married	0.80 (0.60–1.08)	1.83 (1.27–2.62)**
Employment	No	1.00	1.00
	Yes	0.46 (0.37–0.58)***	0.89 (0.67–1.20)
Food insecurity	None	1.00	-
	Mild-to-moderate	0.95 (0.80–1.14)	-
	Severe	0.91 (0.63–1.32)	-
Health insurance	No	1.00	1.00
	Yes	1.27 (1.0–1.6)	1.15 (0.88–1.51)
Assistance	No	1.00	1.00
	Yes	1.24 (1.01–1.53)*	1.14 (0.90–1.46)
Physical activity	Low	1.00	1.00
	Moderate	0.50 (0.41–0.61)***	0.74 (0.58–0.93)*
	High	0.53 (0.40–0.70)***	0.80 (0.58–1.11)
Tobacco	Never	1.00	-
	Current	0.58 (0.46–0.73)***	-
	Quit	0.91 (0.71–1.17)	-
DASH Index	-	1.04 (1.02–1.06)**	-
Add salt/salty sauces to food	Yes	1.00	1.00
	No	1.86 (1.45–2.37)***	1.74 (1.32–2.29)***
Body Mass Index	Normal	1.00	1.00
	Overweight	1.30 (0.95–1.77)	1.20 (0.84–1.72)
	Obese	2.13 (1.60–2.83)***	1.69 (1.20–2.38)**
Other co-morbidities	No	1.00	1.00
	Yes	5.45 (4.51–6.59)***	4.47 (3.64–5.49)***
Mental illness	Minimum	1.00	1.00
	Mild	1.31 (1.02–1.68)*	1.09 (0.81–1.45)
	Moderate-severe	1.48 (1.15–1.90)**	1.14 (0.86–1.52)
Self-rated health	Very good/good	1.00	-
	Half/half	1.88 (1.54–2.31)***	-
	Not good/not good at all	2.92 (2.28–3.72)***	-

In the adjusted model, the variables adjusted for are those with specified adjusted OR value.

*OR* odds ratio; *95% CI* confidence interval; *DASH* Dietary Interventions to Stop Hypertension.

*p < 0.05, **p < 0.01, ***p < 0.001.

Table 5 presents crude and adjusted odds of having controlled hypertension compared to those with uncontrolled hypertension. In the adjusted model, significantly higher odds of controlled hypertension were found among those who were female, mild-to-moderately food insecure, obese, had other co-morbidities, had received all medications for high blood pressure over the past year, did not use traditional home remedies for their blood pressure, and never or rarely added salt or salty sauces to food.

**Table 5:** Correlates of controlled hypertension in Gaza.

Variable	Categories	OR (95% CI) (*N* = 1833)	Adjusted OR (95% CI) (*N* = 1711)
Age	40–49 years	1.00	1.00
	50–59 years	0.79 (0.59–1.06)	0.83 (0.60–1.14)
	60–69 years	1.04 (0.78–1.39)	1.02 (0.74–1.40)
	≥70 years	1.12 (0.81–1.54)	0.96 (0.67–1.37)
Refugee status	Non-refugee	1.00	-
	Refugee	1.12 (0.91–1.37)	-
Sex	Male	1.00	1.00
	Female	1.34 (1.11–1.61)**	1.30 (1.05–1.62)*
Governorate	Khan Yunis	1.00	-
	North Gaza	1.02 (0.74–1.42)	-
	Gaza	1.10 (0.82–1.47)	-
	Rafah	1.34 (0.94–1.90)	-
	Deir Al Balah	1.04 (0.75–1.45)	-
Education	Basic	1.00	-
	Intermediate	0.89 (0.69–1.15)	-
	Secondary or higher	0.94 (0.73–1.21)	-
Wealth quintiles	Poorest	1.00	-
	2	1.16 (0.85–1.57)	-
	3	1.22 (0.90–1.66)	-
	4	1.08 (0.79–1.45)	-
	Richest	1.33 (0.99–1.78)	-
Crowding Index	<= 1 person/room	1.00	-
	2–3 people/room	0.84 (0.67–1.04)	-
	>3 people/room	0.67 (0.46–0.96)**	-
Locality	Non-camp	1.00	-
	Camp	0.81 (0.63.1.04)	-
Marital status	Not married	1.00	-
	Engaged/Married	1.06 (0.79–1.42)	-
Employment	No	1.00	1.00
	Yes	0.62 (0.46–0.83)**	0.78 (0.55–1.09)
Food insecurity	None	1.00	1.00
	Mild-to-moderate	0.98 (0.81–1.18)	1.08 (0.89–1.40)
	Severe	0.52 (0.34–0.82)**	0.57 (0.35–0.93)*
Health insurance	No	1.00	-
	Yes	1.07 (0.82–1.38)	-
Assistance	No	1.00	1.00
	Yes	0.86 (0.69–1.08)	0.80 (0.63–1.03)
Receives blood pressure medication over the past year	Some/none	1.00	1.00
	Yes, all	2.38 (1.49–3.79)***	2.19 (1.36–3.53)**
Service provider for hypertension care	UNRWA	1.00	-
	Government	0.87 (0.71–1.07)	-
	Does not follow up	0.70 (0.36–1.35)	-
Source of hypertension medication	UNRWA	1.00	-
	Government	0.85 (0.70–1.03)	-
Physical activity	Low	1.00	-
	Moderate	0.84 (0.67–1.06)	-
	High	1.00 (0.73–1.37)	-
Tobacco	Never	1.00	-
	Current	0.87 (0.66–1.14)	-
	Quit	0.85 (0.65–1.11)	-
Traditional home remedies	Yes	1.00	1.00
	No	1.35 (1.08–1.69)**	1.26 (1.00–1.59)*
DASH Index	-	1.00 (0.98–1.02)	-
Add salt/salty sauces to food	Yes	1.00	1.00
	No	1.24 (0.91–1.67)	1.21 (0.88–1.65)
Body Mass Index	Normal	1.00	1.00
	Overweight	0.74 (0.50–1.09)	0.66 (0.44–1.00)
	Obese	0.73 (0.51–1.05)	0.61 (0.41–0.90)*
Other co-morbidities	No	1.00	1.00
	Yes	1.10 (0.89–1.35)	1.12 (0.89–1.40)
Mental illness	Minimum	1.00	-
	Mild	0.91 (0.70–1.18)	-
	Moderate-severe	1.13 (0.88–1.46)	-
Self-rated health	Very good/good	1.00	-
	Half/half	0.96 (0.77–1.19)	-
	Not good/not good at all	1.08 (0.86–1.14)	-

In the adjusted model, the variables adjusted for are those with specified adjusted OR value.

*OR* odds ratio, *95% CI* confidence interval, *DASH* Dietary Interventions to Stop Hypertension.

*p < 0.05, **p < 0.01, ***p < 0.001.

**Fig. 2:**
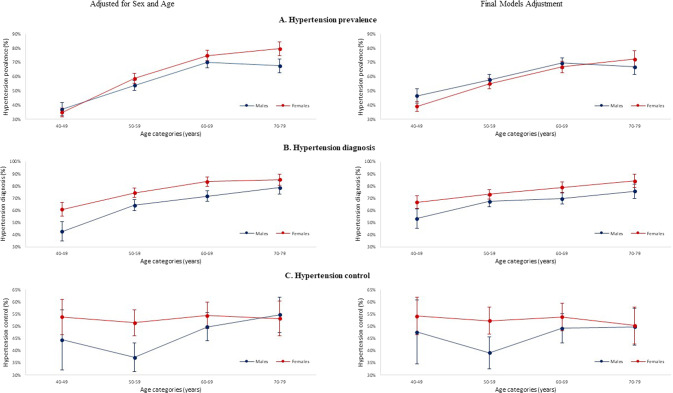
Effect modification of age and sex on the hypertension cascade. Effect modification of age and sex is shown on the prevalence (**A**), diagnosis (**B**) and control (**C**) of hypertension.

Figure 2 shows the effect modification of age and sex on hypertension prevalence, diagnosis and control. With the final model adjustments, we observe that males of younger age have higher risk of having hypertension than women, but the trend reverses for females over 70 years old. Conversely, females in all age categories have a higher chance of being diagnosed with hypertension than their male counterparts, and for all those diagnosed with hypertension, females also have an overall higher chance of controlling their blood pressure than their males counterparts (however the limited sample size increased the confidence interval in this group).

## Discussion

To the best of our knowledge, this is the first representative, comprehensive household survey which explores the hypertension cascade in the Gaza Strip. Unlike other studies conducted in Gaza which were conducted at health facility level and focused on certain aspects of hypertension such as control or risk factors, it focuses on assessing hypertension cascade in the community. Surveying people at their households allowed for the inclusion of marginalized people who face barriers in accessing health care. Our 96.6% response rate is in spite of the difficulties faced with political uncertainties and COVID-19 restrictions, and is similar to other household surveys conducted in the Gaza Strip [3] and the demographics of our sample closely match the most recent national census conducted in 2017 [3]. We therefore have confidence in the representativeness of our sample and the external validity of our results and believe that these data provide an accurate reflection of the population burden of hypertension. Conclusions drawn from the paper can be inferred to analogous conflict-affected settings which usually lack such evidence. Moreover, findings of this study can help policy makers and actors to identify key points of intervention required to control hypertension at the prevention and management fronts in this challenging setting and also in humanitarian context.

Our findings show that over half of the sample had hypertension (using the cut off points of ≥140/≥90 mmHg), of whom about 70% reported being diagnosed and a third found to have their hypertension controlled. Projecting our results onto the full Gazan population, we estimate that the Gaza Strip has ~181,131 residents with hypertension, of whom 51,621 have not yet been diagnosed and 65,069 (including those undiagnosed) do not have their hypertension controlled. Our estimates of hypertension prevalence (56.5%) are over double previous estimates drawn from health facility data in the Gaza Strip [19] which report a prevalence of 27.1% among refugees aged 40 years and older and reflect substantial unmet need across the cascade of care and considerable scope to improve hypertension detection and management across the healthcare system. It is essential to scale up the current facility-based screening program and to launch outreach screening programs to reach more people in the community, including those who do not physically visit health facilities due to various accessibility barriers. Also, it is important to advocate to include hypertension community screening in the PCBS periodic household surveys in order to identify undiagnosed cases.

Our findings underscore important psychosocial and contextual correlates associated with the development of hypertension and suggest that these particularly vulnerable categories need to be proactively targeted in health promotion and screening programs as they are more at risk for developing hypertension (Table 3). Beside paying greater attention to those who are at greater risks for developing hypertension in health promotion and screening programs, including those who are older in age, refugees, those with lower educational attainment, ex-smokers, those who are overweight, or had other co-morbidities or mental illness, it is important to address the root causes of their vulnerabilities through launching a multi-sectoral prevention strategy to improve socioeconomic and environmental determinants of health.

We have identified an unprecedented burden of hypertension in this vulnerable population exposed to protracted conflict, Israeli occupation, profound poverty, psychological distress, and substandard living conditions. Although the prevalence of hypertension is going down world-wide [2], it is going up in Gaza [2], possibly due to the compounded socioeconomic and political challenges Gazans face for several decades. Conflict is consistently associated with hypertension in the academic literature [2] and this poses challenging barriers to overcome primary and secondary hypertension prevention efforts in the Gaza Strip. Opportunities for physical activity and nutritious food [4, 6] are severely restricted in a setting like the Gaza Strip where the population is highly dependent on social assistance to meet their healthcare and wider living needs. Men perform more poorly across the cascade of care than women, which may partly reflect greater interactions by women with the healthcare system for routine check-ups in reproductive and child health [19, 20]. Also, the opportunity for being diagnosed is higher among refugees who are served by UNRWA which provides free-of-charge and better regulated services [8].

Our findings identify that the undiagnosed Gazan population were predominantly younger, male, non-refugees, who are physically inactive, obese, and have poor mental health. These subgroups should be given priority in screening programs and measures targeting their health seeking behaviors. The revealed poor control status among people with hypertension is consistent with the literature which shows similar results in Gaza [20, 21]. It is worth noting that controlling hypertension is an outcome of interplay of many factors including individual, contextual and health services related factors and requires concerted multi-faceted actions at different levels. These include improving the quality of services, better adherence of patients with treatments, encompassing pharmaceutical and nonpharmaceutical approaches aimed at life-style changes together with adequate follow up and monitoring [22]. Addressing the social and conflict related determinants of poor health in this population is also crucial.

The worryingly high percentage of undiagnosed hypertension and the poor control status have substantial short- and long-term adverse complications for the residents of the Gaza Strip. However, increasingly limited financial resources and the ongoing war-like context has created tremendous constraints to accessibility and implementation of community-based screening programs [4]. Investing in comprehensive but cost-effective NCD management initiatives that mix pharmaceutical and non-pharmaceutical approaches that aim to strengthen early detection and timely treatment is critical [23]. Affordable and effective treatments for hypertension have large benefits on coronary heart disease, stroke, and renal disease and are important strategies to mitigate the burden of NCDs more generally in Gaza. Given that the majority of people diagnosed with hypertension were receiving medications demonstrates that the fragmented Gazan healthcare system can procure and prescribe anti-hypertensive medication, but this requires improvements in effectiveness. It is, however, a testimony to the resilience of organizations like UNRWA who maintain services in the face of tremendous political instability and uncertainty [24]. Major challenges may therefore center on primary prevention through behavior risk factor modification, screening methods to better detect the large numbers of undiagnosed people with hypertension, and high levels of adherence from both healthcare providers and patients to maintain good control.

We have shown that insights into hypertension prevalence and risk factors are characterized by intersecting, long-term compounded vulnerabilities resulting from protracted crisis and political turbulence, economic hardship, weak services provision, and inadequate governance which combined have exacerbate health vulnerabilities. The study is, however, not without its limitations. The cross-sectional design precludes causal inferences and the self-reported nature of many variables, including reporting a hypertension diagnosis, may be prone to recall or social desirability biases. However, we ensured to prioritize validated survey questions to minimize such biases and believe that these results will play a crucial role in the public health and health service approach to hypertension in the Gaza Strip. Also, menopause, a risk factor for cardiovascular disease, was not adjusted for in our analysis. Moreover, because this survey relied on self-reported responses, we did not collect data about the types of anti-hypertension medication and the control rate of different medications.

This study has a plethora of important research and policy implications. The richness of the data collected will provide the basis for our ongoing work to model reductions in disease burden and their cost-benefits, which is vital in the context of desperate financial challenges to healthcare in Palestine. The study provides insights into NCD prevalence and risk factors in areas characterized by intersecting, long-term compounded vulnerabilities resulting from protracted crisis and political turbulence, economic hardship, weak services provision, and inadequate governance which combine to exacerbate health vulnerabilities including the development of NCDs. Although the prevalence reported in this study for the different stages of the cascade of care seem superior than the worldwide and region trends recently reported [25, 26] actions are needed to generate better prevention of hypertension and improvement in its diagnosis and control [27]. Controlling hypertension and NCDs more broadly requires concerted multi-faceted actions at different levels, some of these much beyond the traditional boundaries of the health care system. Health promotion policies and programs that tackle the risk factors of hypertension, together with strategic surveillance and monitoring, is especially important given that the current basic package of health services is excessively curative. It is also essential to scale up NCD screening programs and implement proactive and community-level targeted outreach. Such targeting can be guided by our model estimates e.g., targeting younger, male refugees who are overweight and have poor mental health, and can be used to evaluate existing programs for their impact on inequalities. This study’s data and further analytical modeling techniques, modeling potential impacts of different interventions to reduce NCDs, can be used as ways to target the most cost-effective policies and interventions to reduce the development of hypertension and improve its screening and management.

Further research can also elicit the extent of out-of-pocket spending and its implications for healthcare delivery and quality. It is essential to study in more depth the underlying barriers behind the lack of hypertension control, to check for guideline implementation, and to set multi-sectoral programs to address these barriers.

### Summary table

#### What is known about the topic?

- Hypertension is now a leading cause of death and disability in protracted conflict settings, including Gaza.
- Little is known about the prevalence and management of hypertension in Gaza.

#### What this study adds

- This study provides the first robust, validated, and representative measure of hypertension in Gaza.
- Of 4756 respondents, 56.5% had hypertension of which 71.5% had been diagnosed.
- Profound sociodemographic inequalities exist in the prevalence and control of hypertension in this population.

## Data Availability

De-identified participant data will be made available, upon reasonable request, following assessment from the corresponding authors. Datasets will be made available following publication of this manuscript. To request data, please contact Dr. Bassam Abu Hamad at babuhamad@staff.alquds.edu.
